# Sinus Floor Elevation With Platelet‐Rich Fibrin From Horizontal Centrifugation and Xenograft: Randomized Clinical Trial

**DOI:** 10.1111/cid.70093

**Published:** 2025-10-02

**Authors:** Gabriel Guerra David Reis, Ricardo Júnior Denardi, Sergio Luís Scombatti de Souza, Pedro Henrique Felix Silva, Flávia Furlaneto, Richard J. Miron, Carlos Fernando Mourão, Michel Reis Messora

**Affiliations:** ^1^ Department of Oral & Maxillofacial Surgery and Periodontology Ribeirão Preto School of Dentistry, University of São Paulo—USP Ribeirão Preto Brazil; ^2^ Department of Periodontology University of Bern Bern Switzerland; ^3^ Department of Periodontology Tufts University School of Dental Medicine Boston Massachusetts USA

**Keywords:** bone graft, deproteinized bovine bone, H‐PRF, maxillary sinus augmentation, platelet concentrates, platelet‐rich fibrin, xenogeneic

## Abstract

**Objective:**

To evaluate the effects of maxillary sinus augmentation (MSA) using deproteinized bovine bone material (DBBM) combined with or without platelet‐rich fibrin obtained by horizontal centrifugation (H‐PRF) after a short healing period of 4 months.

**Materials and Methods:**

Thirteen patients underwent bilateral two‐stage MSA using a split‐mouth model. Each side was randomly assigned to receive DBBM alone (control group) or DBBM + H‐PRF (test group). Bone tissue samples were harvested 4 months after implant placement and evaluated using microcomputed tomography (micro‐CT), as well as histological and histomorphometric analyses. Data were statistically analyzed using paired *t*‐tests (Wilcoxon signed‐rank test; *p* < 0.05).

**Results:**

Histomorphometric analysis demonstrated higher amounts (*p* < 0.05) of newly formed bone in the DBBM + H‐PRF group compared to the control group (51.33% ± 6.17% versus 45.68% ± 6.65%, respectively). Micro‐CT also revealed significantly higher bone volume (30.38% ± 11.24% and 21.38% ± 9.83%, respectively) and connectivity density (4485 ± 1469 and 2562 ± 1271, respectively) in the DBBM + H‐PRF group than in the DBBM‐alone group (*p* < 0.05).

**Conclusions:**

Compared with DBBM alone, maxillary sinuses augmented with H‐PRF combined with DBBM exhibited improved qualitative and quantitative new bone formation after 4 months of healing. However, the effects on the long‐term survival and early stability of dental implants remain unknown and warrant further investigation with long‐term follow‐up.

## 1 Introduction

The posterior maxillary region presents a challenge for dental professionals due to bone remodeling after tooth loss and maxillary sinus pneumatization [1, 2, 3]. In regions with residual bone height (RBH) less than 4 mm, bone regeneration can be achieved through maxillary sinus floor elevation techniques [4, 5]. This procedure is a predictable therapeutic option [6] with high clinical success rates for the future dental implant osseointegration [7, 8, 9].

Several principles should be considered when selecting grafting material for maxillary sinus augmentation (MSA). These include osteoconductive potential (the ability to serve as a scaffold for new bone formation), osteoinductive potential (the ability to recruit and induce new bone formation), and osteogenic potential (the ability to provide living progenitor cells that facilitate new bone formation) [10, 11, 12]. Compared to other graft types, autogenous bone possesses innate osteogenic and osteoinductive properties. It can promote significant levels of newly formed bone (NFB) within short healing periods [11]. However, data from both clinical and animal models have consistently demonstrated that autografts typically resorb more quickly, with fewer residual graft particles being observed [13]. Therefore, their clinical applicability in MSA is often less favorable due to their unpredictable remodeling outcomes. Furthermore, additional drawbacks include the risk of second‐donor site morbidity, limited donor site availability, and increased surgical time/access requirements.

Alternative grafting options have therefore been explored to enhance graft material stability and minimize the need for a second surgical site [2, 11]. In this context, deproteinized bovine bone mineral (DBBM) derived from xenogeneic bone has proven to be an effective clinical alternative despite lacking osteoinductive and osteogenic potential, due to its long‐term stability as a bone graft [1, 2, 11, 14, 15].

Some approaches proposed to potentially reduce healing time and enhance the bone formation in MSA include the combination of DBBM with certain biologic agents, such as recombinant human bone morphogenetic protein‐2 (rhBMP‐2) and recombinant human platelet‐derived growth factor (rhPDGF). However, these therapies have some limitations, including high costs, potential adverse effects, highly heterogeneous clinical outcomes, and the requirement for strict regulatory approval for routine clinical use [16, 17, 18, 19, 20]. An alternative to these agents is platelet‐rich fibrin (PRF) [21], an autologous and low‐cost compound with favorable clinical applicability.

PRF is a cellular framework composed of platelets and leukocytes [22]. It promotes the acceleration of new bone formation through the release of growth factors over time [23, 24, 25, 26]. It has also been shown to enhance vascularization of bone tissue, with known effects on osteoblast differentiation [27, 28], reduce tissue inflammation, and exhibit antimicrobial properties [29, 30, 31]. However, previous studies involving the combination of PRF with xenogeneic biomaterials during MSA with longer healing periods (6–8 months) have shown limited advantages in terms of NFB levels compared with DBBM alone [2, 3]. Well‐designed clinical studies are often necessary to properly evaluate the effects of PRF on bone healing when combined with xenografts, including the potential to reduce healing times during MSA procedures [3, 32, 33].

Various centrifugation protocols have been proposed since the original leukocyte and platelet‐rich fibrin (L‐PRF) was developed, which has limited clinical comprehension and hindered comparisons of its effects across studies [24, 34, 35, 36]. Establishing optimal protocols is crucial to enhance regenerative outcomes in patients [2, 37]. One advantage of horizontal centrifugation (H‐PRF) is its ability to produce PRF clots with a more uniform structure and higher cell concentrations [34, 35]. Compared with other PRF protocols, this method can achieve higher concentrations and distributions of platelets/leukocytes throughout the matrix, thereby enhancing the antibacterial potential [29, 34, 35, 36, 38]. In a recent systematic review comparing horizontal centrifugation of PRF versus fixed‐angle centrifugation, 84.6% of studies favored horizontal centrifugation across various preclinical and clinical models, while 15.4% did not differ [39]. None of the studies favored fixed‐angle centrifugation, suggesting it as the more optimized/favorable method for producing PRF [40].

A recent preclinical study demonstrated that combining H‐PRF with DBBM in rabbits' sinus augmentation resulted in greater vertical bone gain than DBBM alone [41]. Furthermore, our previous work [26] demonstrated that H‐PRF promoted greater bone formation in rat calvaria critical‐size defects compared with blood clot alone. This study also demonstrated that H‐PRF promoted greater neoformation of bone than PRF obtained through other fixed‐angle centrifugation protocols [26].

Given the current lack of well‐designed clinical studies, this randomized clinical trial (RCT) aimed to evaluate the combination of H‐PRF with DBBM for sinus augmentation after a shortened healing period of 4 months using a split‐mouth design.

## 2 Materials and Methods

### 2.1 Ethical Aspects and Study Design

This prospective, double‐blind, controlled, randomized clinical trial was conducted in the Department of Oral and Maxillofacial Surgery and Periodontology at the Ribeirão Preto School of Dentistry, University of São Paulo (FORP‐USP). The study was approved by the institution's Ethics Committee, under the number CAE: 29428820.3.0000.5419 on March 16, 2021. The authors adhered to the Declaration of Helsinki (1964, last updated in 2013) while conducting this research with human participants. Moreover, all participants were required to sign an informed consent form before their enrollment in the study.

This research was registered on the ClinicalTrials.gov platform in accordance with the Consolidated Standards of Reporting Trials (CONSORT) guidelines [42] with the ID number NCT05957705 and was conducted from March 2021 to July 2023.

### 2.2 Sample Size Calculation

The sample size was calculated to achieve a statistical power of 80% to detect a significant difference of 30% [43] in the changes in bone volume fraction (BV/TV) between the groups evaluated, using a 95% confidence interval (*α* = 0.05) and a standard deviation of 6.81% [44] as the primary outcome. A total of 13 patients were required, accounting for a dropout and complication rate of 20%.

### 2.3 Patient Selection and Randomization

Thirteen patients, without distinction by sex, aged between 48 and 74 years, treated at the School of Dentistry of Ribeirão Preto—USP, were included in this study. Following the inclusion and exclusion criteria described below and after signing the informed consent, patients were identified by a randomized numerical allocated code (Excel, Microsoft Corporation, Redmond, USA) generated by an independent party not involved in the clinical intervention. Using a split‐mouth design, and prior to the reconstructive procedures, each patient's sinuses were randomly assigned to receive one of the following procedures:

- Control Group—Maxillary sinus augmentation with DBBM (Bio‐Oss Large; Geistlich AG, Wolhusen, Switzerland).
- Experimental Group—Maxillary sinus augmentation with a combination of deproteinized bovine bone graft (DBBM) (Bio‐Oss Large; Geistlich AG, Wolhusen, Switzerland) and solid horizontal centrifugation‐prepared platelet‐rich fibrin (H‐PRF) + liquid H‐PRF [34].

### 2.4 Inclusion and Exclusion Criteria

The inclusion criteria for this study were as follows: patients whose bilateral edentulous and atrophic regions in the posterior maxilla presented (1) the need for at least one osseointegrated implant placement per maxillary sinus region for rehabilitation purposes; (2) insufficient vertical bone height for dental implant placement, with a maximum remaining height of 4 mm; (3) sufficient horizontal bone thickness for future dental implant placement; and (4) edentulism in the rehabilitated area for at least 6 months, with a comparable anatomical configuration and similar residual bone height.

Patients were excluded if they had any contraindications for dental implant placement, required horizontal bone augmentation, were current or former smokers, were pregnant, had inflammatory or autoimmune diseases in the oral cavity, using immunosuppressants, corticosteroids or bisphosphonates, had a history of malignancy in the last 5 years or received radiotherapy, reported excessive alcohol consumption or had decompensated systemic conditions, had uncontrolled periodontal disease, had insulin‐dependent diabetes, had blood‐related diseases, or had a history of reconstructive bone therapies in the maxillary sinus region or a history of oroental communication.

### 2.5 H‐PRF Preparation

The solid H‐PRF membranes and liquid H‐PRF were prepared following the established standards described previously [34]. Venous access was obtained in the patient's arm or back of the hand using a scalp vein set, and blood collection was performed using vacutainer tubes (BD, Franklin Lakes, NJ, USA). For each patient, eight tubes (9 mL/tube) of blood were collected in two subsequent steps. Initially, a collection was performed in six silica‐coated tubes (BD, Franklin Lakes, NJ, USA) for the solid H‐PRF, followed by a second puncture for collection in 2 plastic, dry tubes (BD, Franklin Lakes, NJ, USA) for the preparation of the liquid H‐PRF immediately prior to the beginning of the surgical procedure. During the preparation of H‐PRF, the six tubes were centrifuged in a horizontal centrifuge (Eppendorf 5702, Germany) at a relative centrifugal force (RCF) of 700 g (RCF‐max) for 8 min. After centrifugation, the H‐PRF membranes were removed using surgical forceps and scissors. The fibrin clots were fully extracted, leaving a narrow layer of the centrifuged red portion. The H‐PRF clots were gently compressed and stored in a sterile metal box (XPression Intra‐Lock, FL, USA). Next, two additional tube collections were performed and subjected to horizontal centrifugation (Eppendorf 5702 centrifuge, Germany) at an RCF of 200 g (RCF‐max) for 8 min to prepare the liquid H‐PRF. The liquid component was then aspirated with a sterile 3 mL plastic pipette and separated for grafting material preparation.

### 2.6 Surgical Procedures

After cone beam computed tomography (CBCT) exams and previous rehabilitation planning, patients underwent reconstructive surgery under local anesthesia (4% articaine with 1:100 000 epinephrine; DFL, Rio de Janeiro, RJ, Brazil) and sterile conditions. One hour before surgery, patients received a preoperative protocol of 2 g of amoxicillin, 10 mg of diazepam, and 8 mg of dexamethasone, followed by a mouth rinse (15 mL) with a 0.12% chlorhexidine gluconate solution. Following previously described surgical protocols [15, 44, 45, 46], surgeries were performed by two experienced and calibrated surgeons. Marking and access to the lateral wall of the maxillary sinus were obtained using an 8 mm active tip neurological drill (Maximus, Brazil), followed by manual detachment with specialized instruments (Sinus Lift Instruments—Kirsch set, Helmut Zepf, Germany). After complete detachment of the sinus membrane, the grafting material was placed inside the maxillary sinus based on group allocation. Randomization of sides was performed during patient selection, and the surgeon was informed only at the time of surgery. Examiners and patients were blinded throughout the study.

**FIGURE 1:**
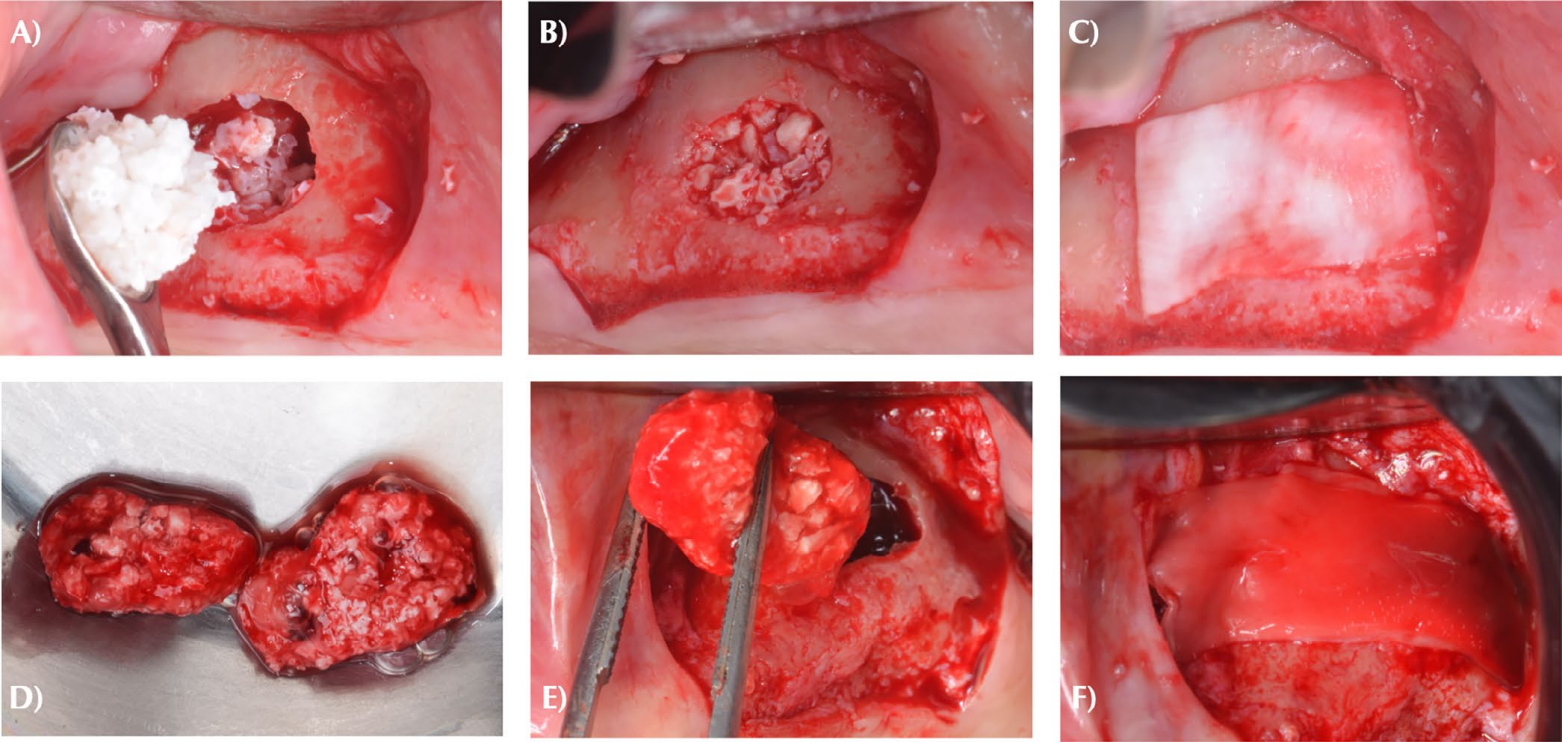
(A) DBBM group—filling of the sinus region with DBBM (Bio‐Oss Large; Geistlich AG, Wolhusen, Switzerland) hydrated in 0.9% saline solution; (B) DBBM group—complete filling of the maxillary sinus; (C) DBBM group—collagen membrane covering the lateral window (BioGide, Geistlich AG, Wolhusen, Switzerland); (D) DBBM + HPRF group—H‐PRF bone block graft after polymerization as a result of the association of solid and liquid DBBM + H‐PRF; (E) DBBM + H‐PRF group—H‐PRF bone block insertion; (F) DBBM + H‐PRF group—H‐PRF membrane covering the collagen membrane (BioGide, Geistlich AG, Wolhusen, Switzerland).

In the control group, MSA was performed using demineralized bovine bone graft (DBBM) (Bio‐Oss Large; Geistlich AG, Wolhusen, Switzerland). The biomaterial was weighed on a precision balance, hydrated in sterile 0.9% saline solution, and placed using appropriate instruments (Sinus Lift Instruments—Kirsch set, Helmut Zepf, Germany). An absorbable collagen membrane (25 × 25 mm) (BioGide, Geistlich AG, Wolhusen, Switzerland) was positioned over the lateral window of the maxillary sinus for complete occlusion (Figure 1).

In the test group, the same MSA DBBM weighing protocol was followed. One solid H‐PRF membrane was fragmented per 0.5 g of DBBM and mixed in a sterile container. Two pipettes of liquid H‐PRF were added to initiate polymerization and form the H‐PRF matrix. The biomaterial was manually shaped to fit the area to be reconstructed and provide access to the sinus window. In the control group, an absorbable collagen membrane (25 × 25 mm) (BioGide, Geistlich AG, Wolhusen, Switzerland) was positioned over the lateral window of the maxillary sinus. A final solid H‐PRF membrane was placed over each collagen membrane before suturing (Figure 1).

In both groups, primary wound closure was performed using 5.0 Soft Blue Nylon sutures (Techsuture, Bauru, São Paulo, Brazil).

Following surgery, patients received postoperative instructions and were prescribed amoxicillin 500 mg every 8 h for 7 days, ibuprofen and arginine 1155 mg (Spidufen, Zambon Laboratórios Farmacêuticos Ltda, São Paulo, Brazil) every 12 h for 5 days, and sodium dipyrone 500 mg every 6 h for up to 7 days. Cautious oral hygiene was recommended, with a gentle rinse of 0.12% chlorhexidine gluconate for 30 s every 12 h for 15 days.

**FIGURE 2:**
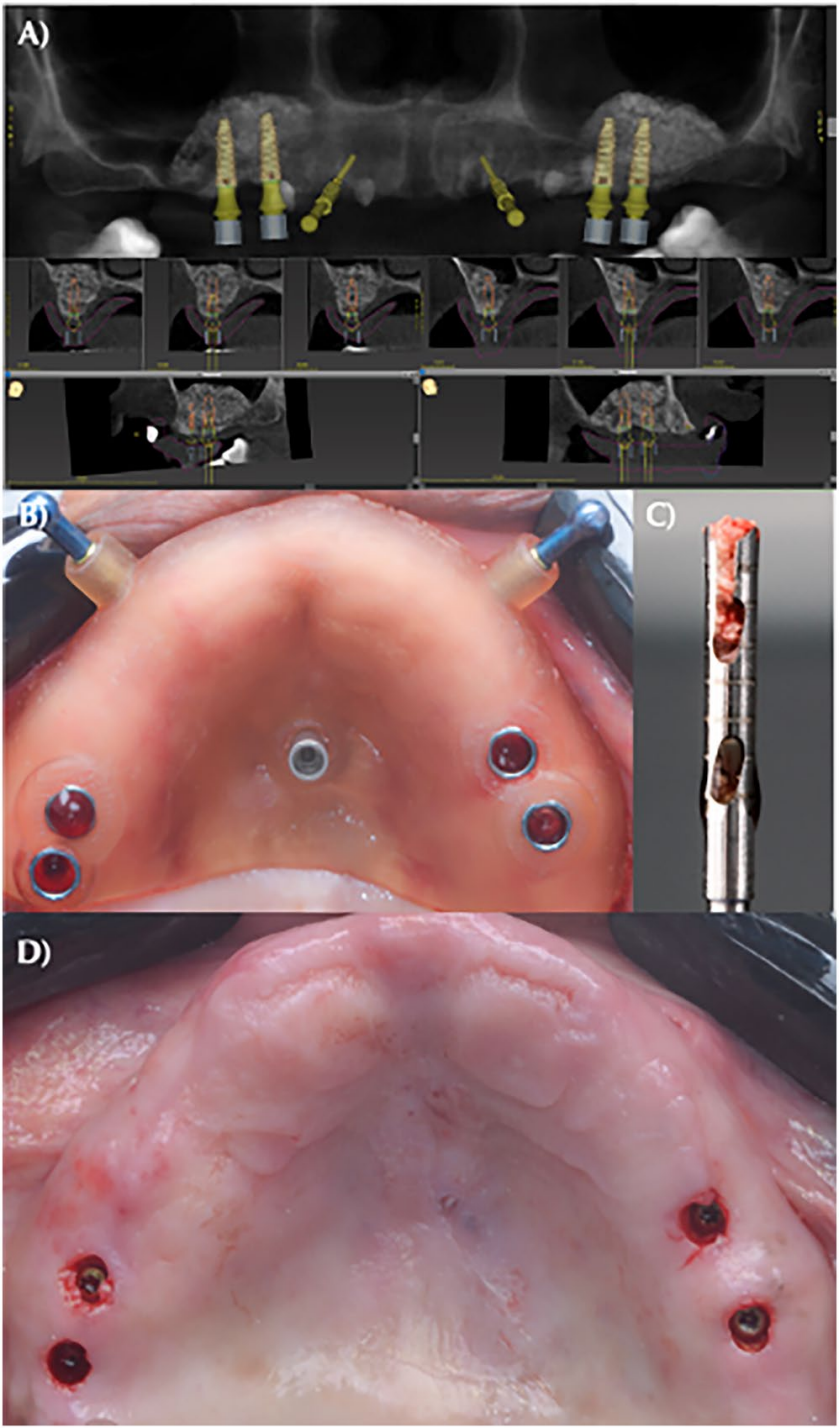
(A) Virtually guided planning using coDiagnostiX (Dental Wings GmbH, Düsseldorf, Germany) for implant placement, selecting the optimal prosthetic and surgical position with remaining cortical bone thickness equal to or less than 4 mm; (B) installation of Easyguide surgical guide (Neodent, Curitiba, Brazil) for implant placement; (C) collection of bone tissue sample using a modified 2.0 mm × 18 mm trephine bur (Wf Cirúrgicos, Barueri, São Paulo, Brazil) for tissue biopsy removal without resistance; (D) clinical aspect after implant placement and cover screw placement.

After 4 months of healing, a calibrated specialist performed new CBCT scans, and virtually guided surgeries were designed (coDiagnostiX, Dental Wings GmbH, Düsseldorf, Germany). Implant placement targeted prosthetically favorable positions according to the patient's needs, with a priority given to grafting areas where the RBH is equal to or less than 4 mm. Virtually guided surgeries (Easyguide, Neodent, Curitiba, Brazil) for implant installation (Helix GM Acqua, Neodent, Curitiba, Brazil) were performed following the same medication protocol as the initial procedure (Figure 2). A surgical punch was used to remove soft tissue, followed by a trephine drill (Wf Cirúrgicos, Barueri, São Paulo, Brazil) with an external/internal diameter of 2.0/1.7 mm and a length of 18 mm guided by precision drill guides at a speed of 180 rpm. Cylindrical biopsies were obtained, followed by appropriate instrumentation with drills prior to implant placement (Figure 2). To ensure comparable biopsy conditions and minimize the influence of anatomical variations, the guided approach precisely dictated the biopsy site selection within areas exhibiting compatible anatomical features and similar residual bone height within the reconstructed region. In cases of two and three implants, the bone biopsies from the most distal and central implants, respectively, were selected for micro‐CT and histometric analyses.

Following a 6‐month healing period after implant placement, patients underwent second‐stage surgery for implant uncovering, followed by prosthetic rehabilitation.

### 2.7 Microcomputed Tomography (Micro‐CT) Analysis

Tissue biopsies were immersed in a 10% formalin solution (pH 7) for 48 h, followed by a 24‐h rinse in running water. The samples were subsequently subjected to microcomputed tomography (micro‐CT) analysis (Skyscan 1172, Bruker, Kontich, Belgium) to generate three‐dimensional images. After scanning, the samples were preserved in EDTA. For image acquisition, a spatial resolution of 7.9 μm was adopted, and the X‐ray generator was operated at an accelerated potential of 60 kV with a current of 165 μA.

Using DataViewer software v.1.4.3 (Skyscan NV, Kontich, Belgium), the three‐dimensional images were rotated to a standard position for analysis, and a region of interest (ROI) based on the remaining cortical bone and a volume of interest (VOI) with a diameter of 1.5 mm and a length of 4 mm was standardized for analysis.

To evaluate the NFB and residual DBBM present in each VOI, two grayscale thresholds (circle) were applied, one with specific parameters for the analysis of remaining biomaterial (MV/TV) (155–250; minimum–maximum) and another for the analysis of newly formed bone (BV/TV) (80–155; minimum–maximum). These grayscale ranges were determined based on histogram analysis of grayscale distribution and validated through visual inspection of cross‐sectional images in correlation with known anatomical landmarks. Upon applying these grayscale thresholds, the visual distinction between native bone, NFB, and residual DBBM based on their density differences allowed for precise ROI positioning.

Using CT‐Analyzer software v.1.13.5.1+ (Bruker, Kontich, Belgium), the following parameters were evaluated by a calibrated and blinded examiner: (a) bone volume fraction (BV/TV); (b) trabecular thickness (Tb.Th); (c) total porosity (Po.Tot); (d) trabecular number (Tb.N); (e) trabecular separation (Tb.Sp); (f) total porosity percentage (Po.Tot); (g) open porosity percentage (Po.Op); (h) connectivity density (Conn.Dn); and (i) material volume percentage (MV/TV). Three‐dimensional rendered reconstructions were obtained from the micro‐CT sections for illustrative purposes.

### 2.8 Histological and Histomorphometric Analyses

After micro‐CT analysis, the samples were decalcified and subsequently embedded in paraffin with their long axis oriented to allow longitudinal sectioning along their entire length. Serial sections with a thickness of 4–5 μm were obtained, and the histological slides representing the most central portion of each biopsy were stained with hematoxylin and eosin (H&E).

Histomorphometric analysis was conducted by a calibrated examiner (G.G.D.R.) using a computer‐based image evaluation system and specific software (LAS EZ version 4.1.0, Leica Microsystems GmbH, Wetzlar, Heidelberg, Germany). One representative histological section per sample was selected and photographed using a trinocular microscope equipped for bright‐field and fluorescence imaging (model DMLB, Leica Microsystems GmbH, Wetzlar, Heidelberg, Germany) with a 1.6× objective and a coupled camera (DFC300FX, Leica Microsystems GmbH, Wetzlar, Heidelberg, Germany).

A standardized region of interest was determined, starting from the end of the patient's residual alveolar bone to the apical third of the section. Analysis was performed by an experienced, blinded examiner using ImageJ 1.45 software (National Institutes of Health, USA). The following parameters were evaluated: percentage of NFB, percentage of soft tissue, and percentage of residual graft. Qualitative histological analysis was conducted by observing selected HE‐stained sections under the same microscope, using 10× objectives coupled to the same camera used in the histomorphometric analysis.

### 2.9 Statistical Analysis

Statistical analyses were performed using GraphPad Prism software (GraphPad Software Inc., v.5.01; San Diego, CA, USA). Data normality was assessed using the Shapiro–Wilk and Kolmogorov–Smirnov tests. A paired *t*‐test was used for histomorphometric analysis. For the micro‐CT analysis, considering the data distribution and variance properties, the Wilcoxon signed‐rank test was applied. A significance level of *p* < 0.05 was set for all tests, and the results were reported both textually and graphically.

## 3 Results

**FIGURE 3:**
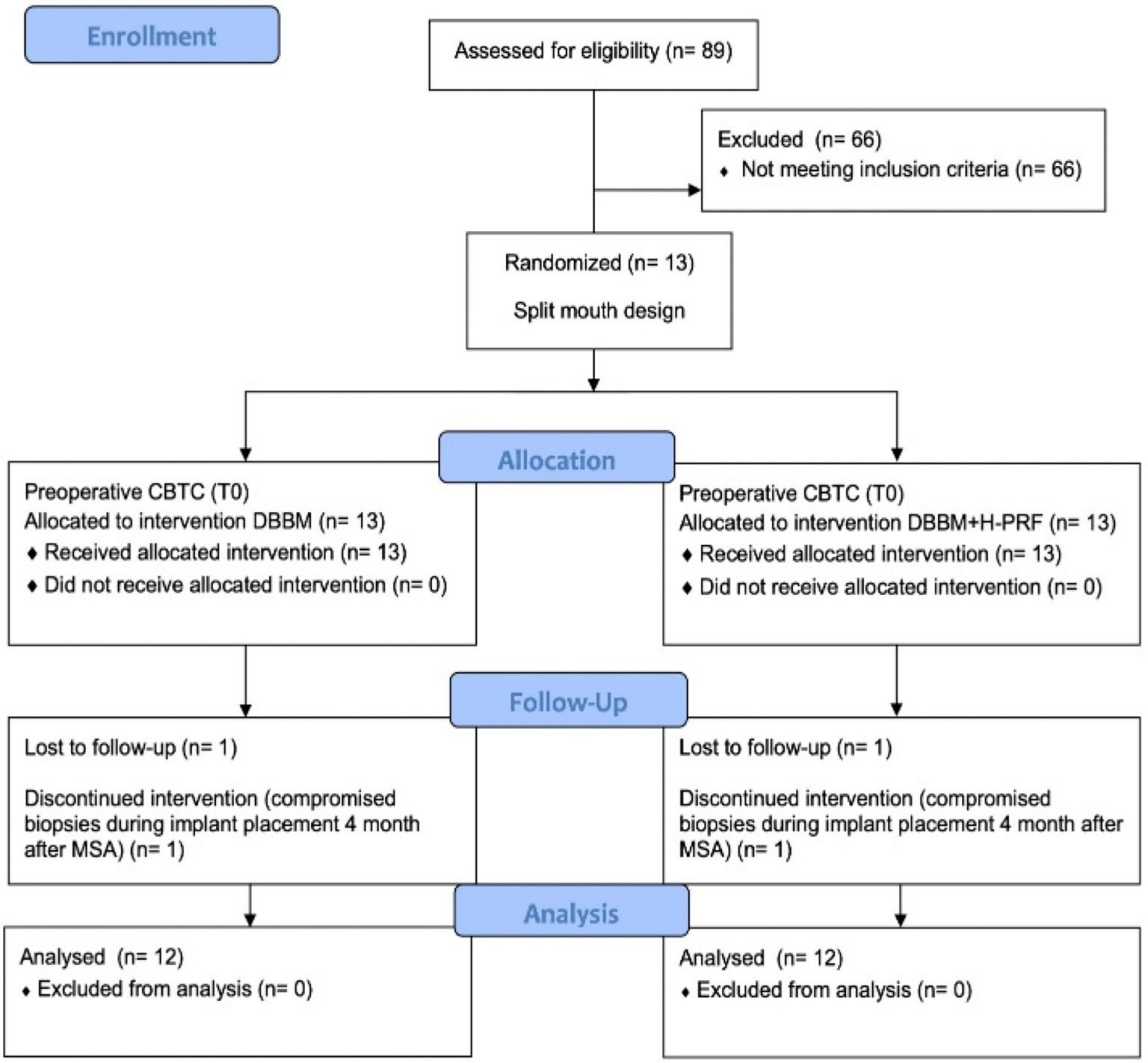
Flowchart of the experimental design. CBCT = cone beam computed tomography; MSA = maxillary sinus augmentation; DBBM = deproteinized bovine bone material; H‐PRF = platelet‐rich fibrin obtained by horizontal centrifugation.

A total of 13 patients, including three males and 10 females, with an average age of 59.23 ± 10.51 years, underwent MSA. Nine patients underwent full‐arch rehabilitations, and 4 underwent partial rehabilitations. The average RBH was 2.6 ± 0.6 mm in the control group (DBBM alone) and 2.1 ± 0.9 mm in the experimental group (DBBM + H‐PRF), with no statistically significant difference between the groups (*p* = 0.418). A total of 49 hydrophilic surface implants (Helix GM Acqua, Neodent, Curitiba, Brazil) were placed in the reconstructed areas 4 months after MSA. Implant diameters ranged from 3.5 to 3.75 mm and lengths from 8 to 11.5 mm. No intraoperative or postoperative complications were reported (Figure 3) (Table 1).

**TABLE 1:** Clinic and demographic data.

Patient	Gender	Age (Y)	Maxillary sinus		Residual bone height (RBH) (mm)		Number of Implants		Edentulism	Prosthetic type
			DBBM	DBBM + HPRF	DBBM	DBBM + HPRF	DBBM	DBBM + HPRF		
A	F	61	R	L	2.5	3	2	2	Total	Multiple | screw‐retained
B	F	64	R	L	2	2	2	2	Total	Multiple | screw‐retained
C	F	64	R	L	2.3	2	3	3	Total	Multiple | screw‐retained
D	F	61	L	R	1	1	3	3	Total	Multiple | screw‐retained
G	F	74	L	R	2	2	1	1	Partial	Unitary | screw‐retained
I	F	74	L	R	3	0.7	2	1	Total	Unitary | screw‐retained
M	M	54	R	L	3	4	2	2	Total	Multiple | screw‐retained
N	F	62	R	L	3.4	2.2	2	2	Total	Multiple | screw‐retained
O	F	52	L	R	2.7	3.5	2	2	Total	Multiple | screw‐retained
P	F	56	R	L	2	3	2	1	Partial	Unitary | cemented‐retained
Q	F	37	L	R	2.9	1.7	1	2	Partial	Unitary | screw‐retained
L	M	49	R	L	3.6	2.3	1	1	Partial	Unitary | cemented‐retained
S	M	62	R	L	3	2.4	2	2	Total	Multiple | screw‐retained

Abbreviations: F, female; L, left; M, male; R, right; Y, years.

The survival rate after 6 months of osseointegration was 100% for the control group and 95.83% for the test group, as evaluated at the time of functional loading of the implants. The only reported failure was attributed to traumatic forces resulting from provisional prostheses in a localized area of implant healing.

### 3.1 Micro‐CT Analysis

Twenty‐six biopsies were collected from the implant installation sites using guided virtual surgery. However, two samples from the same patient were compromised during the surgical procedure due to the tissue fragility and challenging clinical access (Figure 3).

**FIGURE 4:**
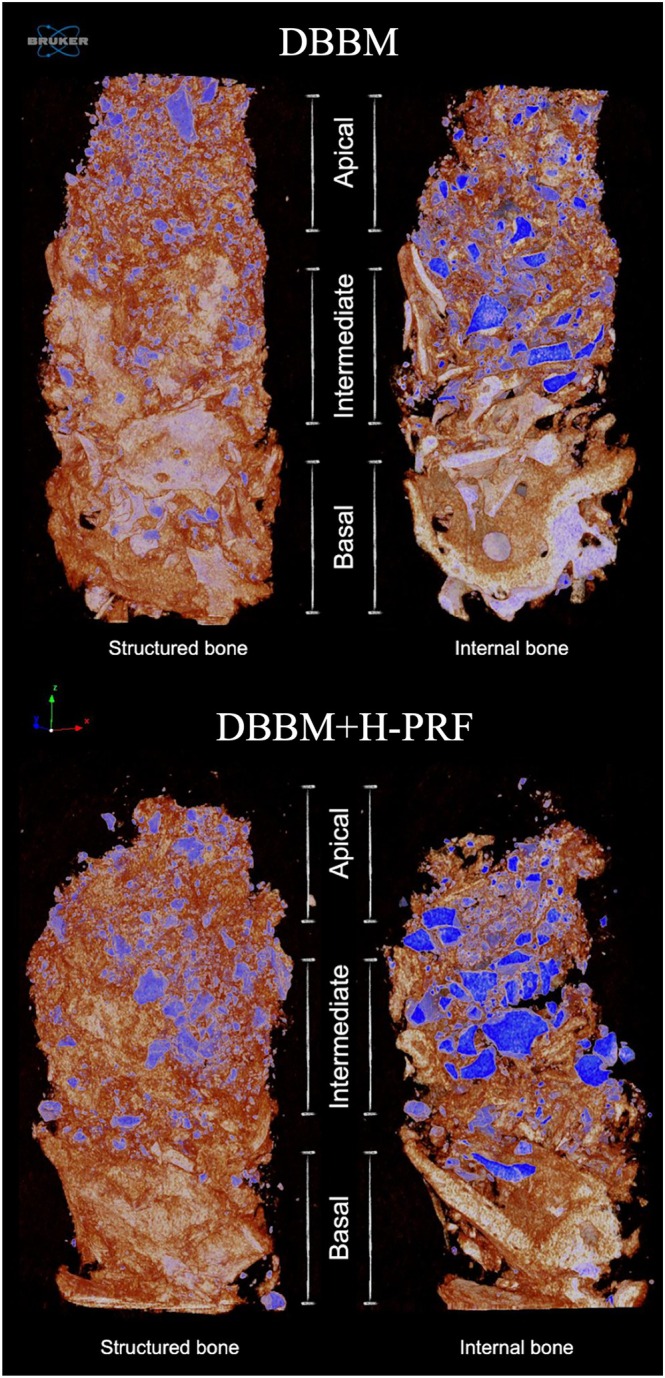
3D Micro‐CT images showing structured bone tissue and internal bone cut of DBBM and DBBM + H‐PRF groups. Remaining bone is depicted in a lighter color in the basal region, NFB is shown in orange throughout the three layers of the biopsies, and the remaining DBBM (xenogeneic bone graft) is depicted in blue in both samples.

A total of 24 samples were analyzed: 12 from the control group and 12 from the experimental group. Three‐dimensional reconstructions revealed remaining cortical bone, residual bone substitute, and NFB in the basal, intermediate, and apical thirds of both groups (Figure 4).

**FIGURE 5:**
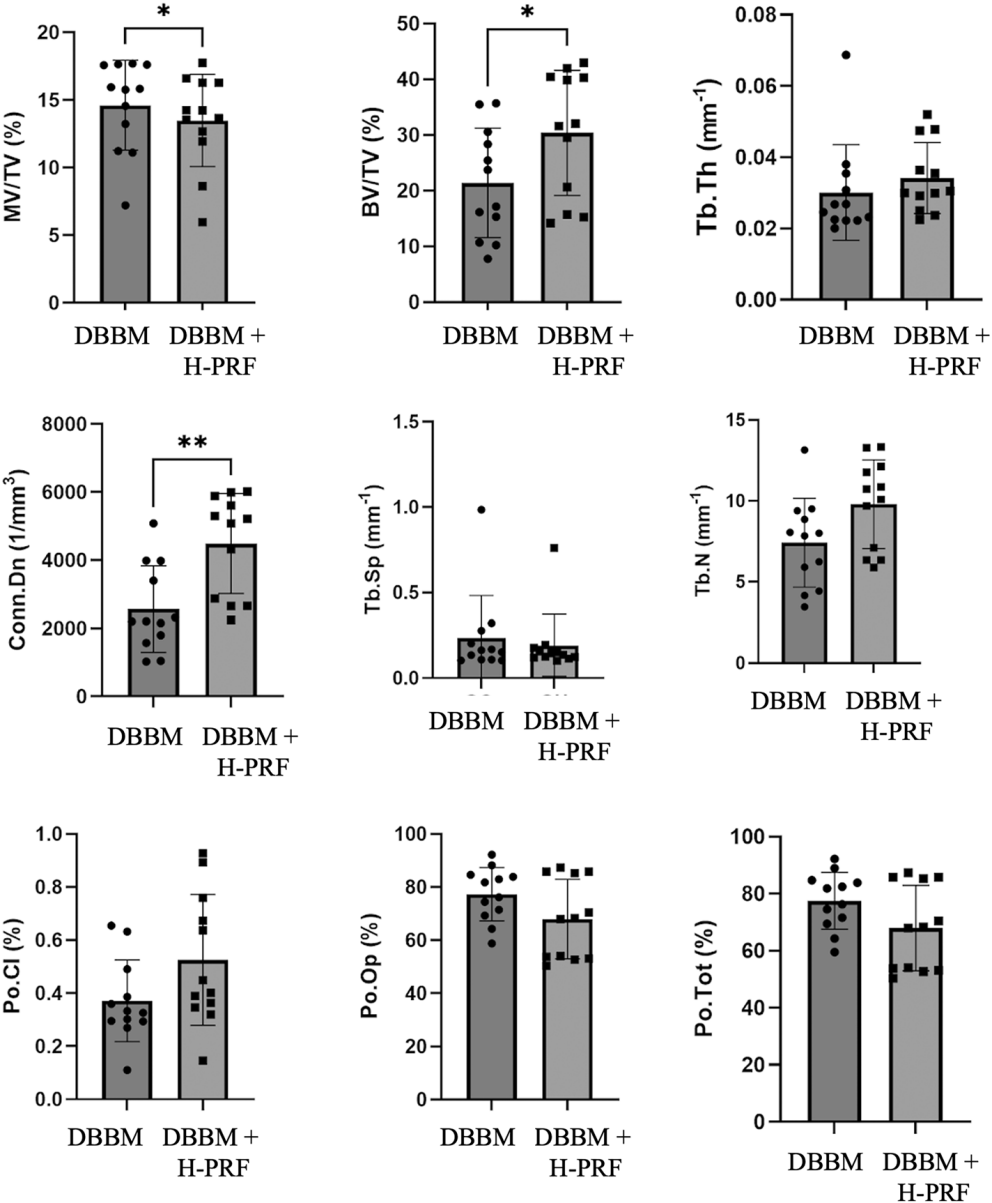
Means and interquartile ranges of the micro‐CT parameters. BV/TV (*p* = 0.0306) and MV/TV (*p* = 0.0359) values demonstrate statistical differences between groups DBBM and DBBM + H‐PRF (*, *p* < 0.05, *t‐*test), as well as in the values of Conn.Dn (*p* = 0.0034) (**, *p* < 0.05, Wilcoxon test). No significant differences were observed between the groups for Tb.Th, Tb.Sp, Tb.N, Po.Cl, Po.Op, and Po.Tot (*p* < 0.05, Wilcoxon test).

The MV/TV values showed higher percentages of remaining bone substitutes in the DBBM alone group than in the DBBM + H‐PRF group (*p* < 0.05). Higher BV/TV values were found in the DBBM + H‐PRF group (30.38 ± 11.24) than in the DBBM alone group (21.38 ± 9.83) (*p* < 0.05), as were higher values of Conn.Dn (DBBM Group = 2562 ± 1271; DBBM + H‐PRF Group = 4485 ± 1469) (*p* < 0.01). There were no significant differences in the values of Tb.N, Tb.Th, Tb.Sp, Po.Tot, Po.Cl, or Po.Op (Figure 5) between the groups.

### 3.2 Histomorphometric Analysis

**FIGURE 6:**
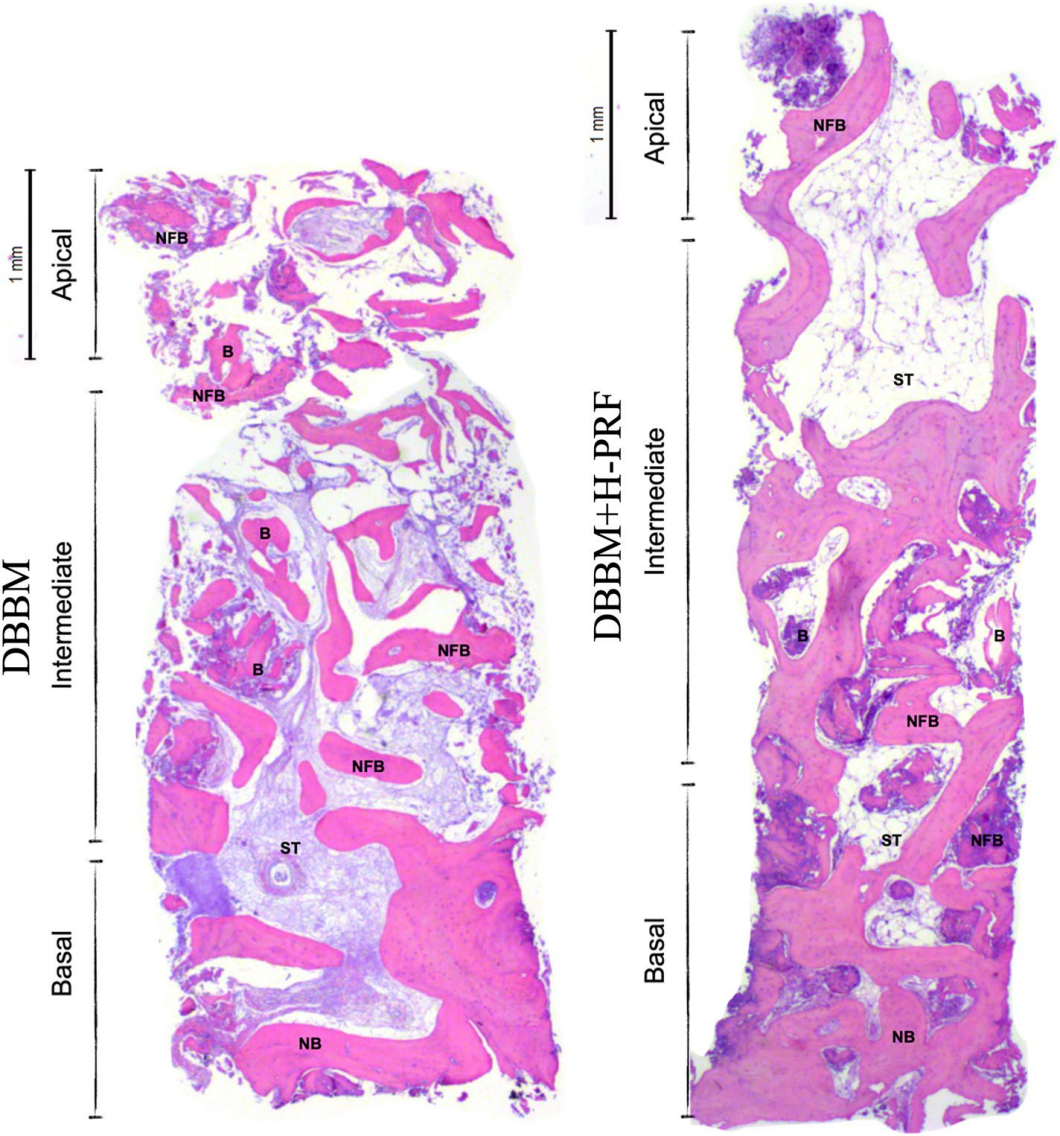
Representative histological sections of groups DBBM and DBBM‐HPRF in the basal, intermediate, and apical areas. NB = Native bone, ST = soft tissue, NFB = newly formed bone, B = deproteinized bovine bone material.

Twenty‐four histological sections were assessed, each of which was representative of an individual sample. In each section, remaining cortical bone, residual bone substitute, and NFB were observed in the intermediate and apical thirds for both groups (Figure 6). The results revealed higher newly formed bone in the DBBM + H‐PRF group (51.33% ± 6.17%) than in the DBBM alone group (45.68% ± 6.65%) (*p* < 0.05). No significant differences in the percentages of soft tissue (26.81% ± 9.08% and 30.21% ± 9.06%, respectively) or residual graft (21.85% ± 8.93% and 24.09% ± 11.79%, respectively) were detected between the groups.

### 3.3 Descriptive Histological Evaluation

DBBM was present throughout the samples, with greater amounts in the intermediate and apical regions in both groups.

**FIGURE 7:**
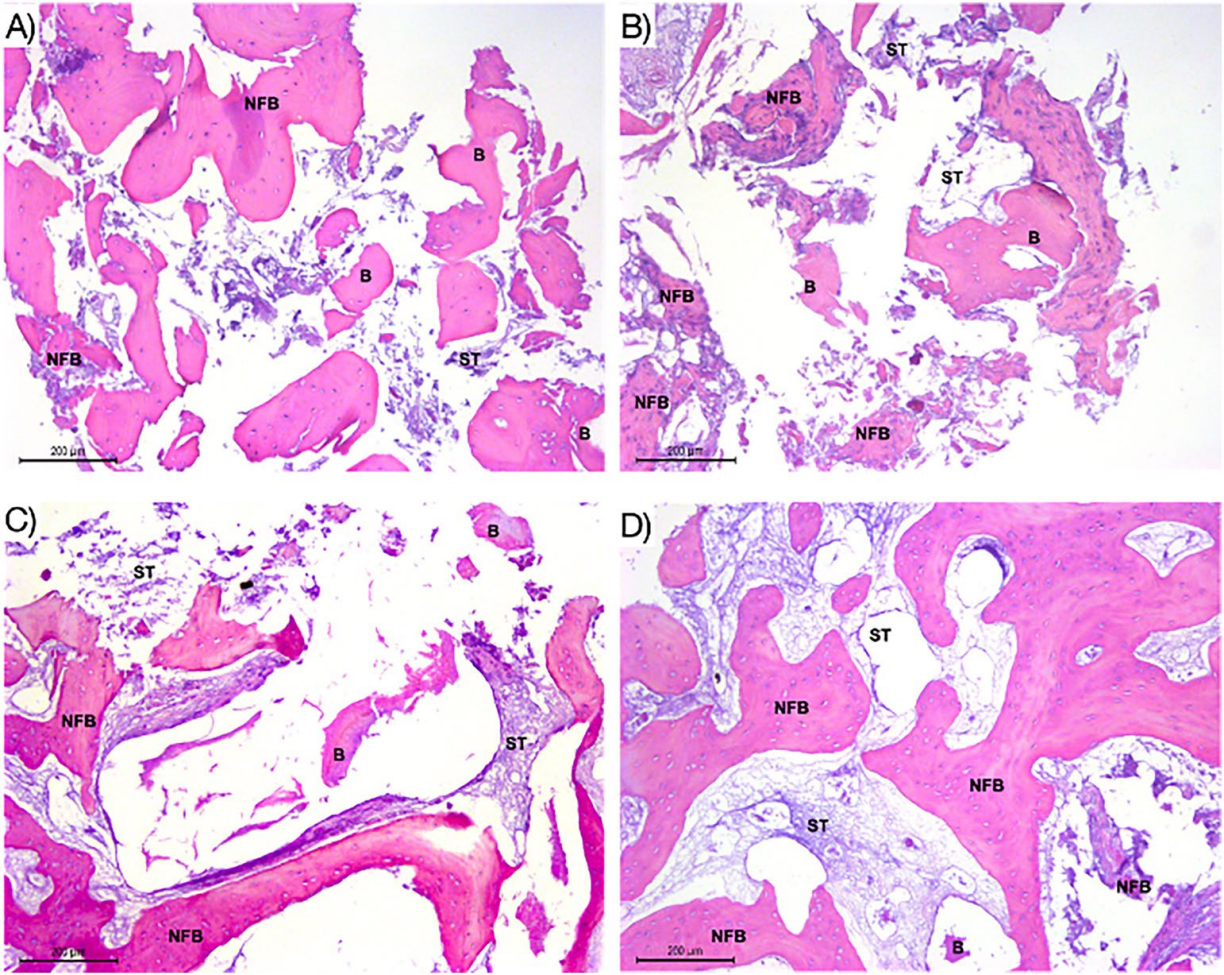
(A) Histological section of the middle‐apical third of the DBBM group demonstrating the presence of biomaterial particles (B) and newly formed bone (NFB). (B) Histological section of DBBM + HPRF group showing widespread presence of osteogenic matrix in NFB, even in more apical areas of the sample, surrounding the biomaterial particles (B). Images (C) and (D) depict the middle third of histological sections of groups DBBM and DBBM + H‐PRF, respectively, demonstrating a noticeable difference in the thickness of NFB between the groups and a difference in the spacing between layers of bone tissue, with the presence of soft tissue (ST) surrounding the biomaterial particles (B).

Even with a shorter healing period, osteoid bone matrix was present in both groups, although with varying amounts among the samples. An osteoid bone matrix was found even in the most apical regions of some sections in both groups. However, it is worth noting that a greater amount of NFB was present in the DBBM group with H‐PRF, particularly in the intermediate region of the samples. In the DBBM + H‐PRF group, a greater proportion of osteogenic areas spread throughout the samples than in the DBBM alone group. In the DBBM alone group, small and more spaced osteogenic areas with fibrous connective tissue between them were observed (Figure 7).

## 4 Discussion

The ideal timing for implant placement following bone regeneration procedures depends on two crucial factors: the required amount of available bone and the maturation of the reconstructed tissue. In this context, the quantity and quality of NFB appear to be critical and relevant factors for evaluating grafting materials. A direct association has been established between a higher volume and tissue density, which leads to better bone‐to‐implant contact (BIC) and, consequently, better implant survival rates [47]. In this study, the group treated with DBBM + H‐PRF demonstrated higher NFB and Conn.Dn values compared to the group treated with DBBM alone at 4 months postoperatively.

Healing time plays a significant role during MSA, particularly when DBBM is applied alone. Due to the absence of osteoinductive growth factors in the biomaterial, healing periods typically exceeding 6 months are generally recommended to achieve sufficient amounts of NFB formation [11]. The optimal duration for bone healing in MSA with staged implant placement is a matter of debate. Insufficient data and studies with low levels of evidence do not support reducing the bone healing phase of MSA with DBBM alone [44]. In a previous study, DBBM implantation in MSA was evaluated at two healing periods. NFB values of 21.47% ± 6.35% and 25.54% ± 7.91% were obtained 5 and 8 months after reconstructive therapy, respectively. Similar patterns of ISQ and implant survival rates were observed in both evaluation periods [44]. According to those authors, MSA with DBBM alone could be reduced to a 5‐month bone healing period; however, the impact on long‐term implant survival is still unknown, and further investigations with long‐term follow‐up are warranted.

Given the vascular and cellular regenerative potential of PRF [23, 27, 28], controlled clinical trials have sought to demonstrate the effectiveness of combining PRF with DBBM [14, 46, 48] to increase NFB formation capacity in MSA and/or reduce the healing time. In this context, histomorphometric analysis revealed NFB patterns ranging from 18.35% ± 5.62% [14] to 21.38% ± 8.78% [46] in MSA with DBBM + PRF at 6 months postoperatively. These values were not significantly different from those obtained in MSA with DBBM alone at these later time points; therefore, such results do not demonstrate any additional benefit of L‐PRF in MSA [2, 3].

Two primary considerations were central to this study. First, protocol improvements and optimization of PRF have resulted in a 4‐fold increase in the number of H‐PRF cell types compared to previous formulations of PRF [39, 40]. Second, while most studies on PRF have focused on later periods during MSA, the benefit might be derived by investigating healing periods of less than 6–8 months to help clarify whether the association of PRF with DBBM fosters additional benefits. To date, only one study has reported higher NFB values in MSA with PRF + DBBM (44.58% ± 13.9%) compared to DBBM alone (30.02% ± 8.42%) [15]. Importantly, in this study, the DBBM + PRF group was evaluated at 4 months postoperatively, and the control group (DBBM only) was assessed at 8 months. Histometric analysis also revealed lower values of residual graft material in the test group (DBBM + PRF) compared to the control group. However, no volumetric analysis (micro‐CT) or quantification of the biomaterial used was reported.

In contrast, the present study evaluated both groups at 4 months postoperatively, enabling a direct comparison of the true benefits of PRF in accelerating healing time in MSA patients. Furthermore, the amount of biomaterial used in each group was controlled and standardized. Similarly, to ensure standardized micro‐CT analysis, MSA biopsy areas were virtually guided for anatomical comparability and similar residual bone height. The PRF preparation protocol was also improved and prepared using horizontal centrifugation (H‐PRF) to promote greater platelet and leukocyte retention, as well as a sustained release of growth factors, thereby enhancing regenerative and healing effects [26, 49].

As a method capable of providing 3D information on all grafted bone samples, as well as a precise analysis of bone tissue characteristics and the possibility of selecting regions of interest (ROI) with similar delimitation in each sample, micro‐CT was utilized as a reliable method for determining NFB in MSA [50]. This technique allows sound measurements of bone volume and the amount of residual bone graft in the reconstructed areas. In this study, the DBBM + H‐PRF group presented higher BV/TV (%) values compared to the control group (DBBA alone; 30.38% ± 11.24% and 21.38% ± 9.83%, respectively). Higher MV/TV (%) values were observed in the DBBM alone group compared to the DBBM + H‐PRF group. Comparison with other studies is limited due to the scarcity of research with shorter healing periods. Using a similar methodology, Liu et al. [44] reported higher BV/TV (%) values (27.97% ± 6.81%) compared to those reported in the present study for the use of isolated DBBM in MSA. However, Liu et al. [44] used a longer healing time for their analyses. Additionally, they reported higher MV/TV (%) values than those observed in the present study when using DBBM alone in MSA. These values may suggest that a greater amount of DBBM was used compared to the present study. Another aspect that may explain the differences in results is the patterns of micro‐CT analysis for delineating newly formed bone and biomaterials. A critical factor that can impact the outcome of micro‐CT analysis is the gray‐level thresholds used. The use of grayscales may lead to overlap between the graft and bone in some locations [51, 52, 53, 54].

It is important to emphasize that a more accelerated bone maturation process was observed in the H‐PRF group compared to the DBBM‐alone group. Higher Conn.Dn values were observed in the DBBM + H‐PRF group than in the control group (4485 ± 1469 and 2562 ± 1271, respectively). These data, combined with the findings of the histopathological analysis, demonstrate the potential of H‐PRF to enhance bone neoformation. A recent preclinical study compared DBBM + H‐PRF to DBBM alone in a New Zealand rabbit model of MSA [41]. Their results demonstrated a higher vertical bone gain of the sinus floor, BV/TV percentage, Tb.Th, and Tb.N, along with lower Tb.Sp, found in the DBBM + H‐PRF group compared with the DBBM alone group. The authors concluded that DBBM + H‐PRF showed greater potential for sinus augmentation by enhancing angiogenesis, bone formation, and bone remodeling [41]. The potential for the isolated use of H‐PRF in bone regeneration was demonstrated in another recent preclinical study [26]. This study evaluated bone neoformation in critical‐size defects (CSDs) created in rat calvaria treated with PRF obtained using three different centrifugation protocols involving high‐ and low‐speed centrifugation, as well as horizontal versus fixed‐angle centrifugation equipment. They found that platelet concentrates produced using horizontal centrifugation yielded platelet concentrates with greater biological potential for bone regeneration in this CSD animal model [26].

A direct comparison of the results from the present study with those obtained using other biologic agents (rhBMP‐2 or rhPDGF) is challenging. There is a scarcity of controlled clinical studies with these agents [20], particularly in MSA and in combination with DBBM. Lin et al. [17] evaluated the effect of rhBMP‐2 on sinus volumetric and histometric changes after sinus floor augmentation compared to conventional non‐biologic bone grafting materials. Their systematic review revealed that the use of rhBMP‐2 in maxillary sinus floor augmentation produced clinical and histometric outcomes similar to those of conventional sinus grafting procedures after a 6–9 month healing period. The use of rhPDGF‐BB was tested in one study, which reported that mineralized tissue formation was approximately 10% higher in the test group after 4–5 months of healing; nevertheless, at 7–9 months, the difference was negligible [19].

In conclusion, maxillary sinuses augmented with H‐PRF combined with DBBM showed superior qualitative and quantitative patterns of new bone formation than those treated with DBBM alone after 4 months of healing. Importantly, the limitations of the present study, including differences in the mesiodistal dimension of the grafted area and the short follow‐up period for dental implants, should be considered. The mesiodistal dimension of the grafted area may have influenced the outcomes, as osteogenic factors originate from the elevated Schneiderian membrane and adjacent pristine bone. The long‐term survival of placed dental implants remains unknown and requires further investigation with long‐term follow‐up. The individual contributions of several factors inherent to the surgical procedure, such as guided surgery for implant installation, types of dental implant surfaces, type of prosthetic rehabilitation, and PRF preparation protocol, should also be investigated in future studies. The inclusion of both volumetric and linear pre‐ and post‐operative CBCT evaluations in these studies would enable a more comprehensive and thorough assessment of the grafted maxillary sinuses. Furthermore, additional studies with longer healing periods are essential to evaluate the effects of H‐PRF on the ability to impact bone formation. Based on the qualitative histological analysis of this study, it can be speculated that the DBBM + H‐PRF group would exhibit a greater quantity of newly formed bone than the DBBM alone group at 6–8 months postoperatively.

## Author Contributions

**Gabriel Guerra David Reis:** conceptualization, methodology, software, data curation, investigation, validation, formal analysis, funding acquisition, visualization, project administration, resources, writing – original draft, writing – review and editing. **Ricardo Júnior Denardi:** conceptualization, methodology, software, data curation, investigation, validation, formal analysis, funding acquisition, visualization, project administration, resources, writing – original draft, writing – review and editing. **Sergio Luís Scombatti de Souza:** conceptualization, methodology, data curation, investigation, validation, formal analysis, supervision, visualization, writing – original draft, writing – review and editing. **Pedro Henrique Felix Silva:** conceptualization, methodology, software, data curation, validation, formal analysis, visualization, project administration, writing – original draft, writing – review and editing. **Flávia Furlaneto:** conceptualization, methodology, software, data curation, validation, formal analysis, writing – original draft, writing – review and editing. **Richard J. Miron:** conceptualization, methodology, data curation, validation, formal analysis, funding acquisition, resources, writing – original draft, writing – review and editing. **Carlos Fernando Mourão:** conceptualization, methodology, data curation, validation, formal analysis, supervision, writing – original draft, writing – review and editing. **Michel Reis Messora:** conceptualization, methodology, software, data curation, investigation, validation, formal analysis, supervision, funding acquisition, visualization, project administration, resources, writing – original draft, writing – review and editing.

## Conflicts of Interest

Richard J. Miron is the founder of Miron Research and Development in Dentistry LLC and holds intellectual property in the production of platelet‐rich fibrin. The other authors declare no conflicts of interest.

## Data Availability

The data that support the findings of this study are available from the corresponding author upon reasonable request.
